# Singular Case of Lactation

**Published:** 1857-09

**Authors:** 


*Singular Case of Lactation.*—A few days since, my attention was called, by a friend, to an old woman who was suckling her grand child. Seven years ago I delivered her of her of last child, which died a few weeks after, and, in womans' parlance, her milk soon dried up. Last Christmas her eldest daughter died, leaving an infant which fell to her charge. As she stated to me, at night the child would take the nipple in its mouth, until she actually began to give an abundant supply of milk, after an interval of more than six years of non-lactation. Any one who has curiosity about the matter, can see this singular case, by going to the kitchen of the late Russell Dudley, diagonally opposite the United States Hotel. —Thomas Johnson, in Virginia Medical Journal.—*Boston Medical Journal.*

